# Photochemical Hydrogen Evolution at Metal Centers Probed with Hydrated Aluminium Cations, Al^+^(H_2_O)_*n*_, *n*=1–10

**DOI:** 10.1002/chem.202103289

**Published:** 2021-11-05

**Authors:** Jakob Heller, Tobias F. Pascher, Christian van der Linde, Milan Ončák, Martin K. Beyer

**Affiliations:** ^1^ Institut für Ionenphysik und Angewandte Physik Universität Innsbruck Technikerstraße 25 6020 Innsbruck Austria

**Keywords:** conical intersections, hydration, multireference calculations, water clusters, water splitting

## Abstract

Hydrated aluminium cations have been investigated as a photochemical model system with up to ten water molecules by UV action spectroscopy in a Fourier transform ion cyclotron resonance (FT‐ICR) mass spectrometer. Intense photodissociation was observed starting at 4.5 eV for two to eight water molecules with loss of atomic hydrogen, molecular hydrogen and water molecules. Quantum chemical calculations for *n*=2 reveal that solvation shifts the intense 3s–3p excitations of Al^+^ into the investigated photon energy range below 5.5 eV. During the photochemical relaxation, internal conversion from S_1_ to T_2_ takes place, and photochemical hydrogen formation starts on the T_2_ surface, which passes through a conical intersection, changing to T_1_. On this triplet surface, the electron that was excited to the Al 3p orbital is transferred to a coordinated water molecule, which dissociates into a hydroxide ion and a hydrogen atom. If the system remains in the triplet state, this hydrogen radical is lost directly. If the system returns to singlet multiplicity, the reaction may be reversed, with recombination with the hydroxide moiety and electron transfer back to aluminium, resulting in water evaporation. Alternatively, the hydrogen radical can attack the intact water molecule, forming molecular hydrogen and aluminium dihydroxide. Photodissociation is observed for up to *n*=8. Clusters with *n*=9 or 10 occur exclusively as HAlOH^+^(H_2_O)_*n*‐1_ and are transparent in the investigated energy range. For *n*=4–8, a mixture of Al^+^(H_2_O)_*n*_ and HAlOH^+^(H_2_O)_*n*‐1_ is present in the experiment.

## Graphical abstract

**A complex sequence of events**, including intersystem crossing to the triplet surface and electron transfer, is involved in photochemical hydrogen evolution in Al^+^(H_2_O)_*n*_. For two water molecules, high‐level calculations reveal a scenario for atomic hydrogen formation in which the system passes through a conical intersection between the two lowest‐lying triplet states, and the hydrogen atom is virtually ejected while the remaining hydrated aluminium hydroxide cation relaxes to its new equilibrium geometry.

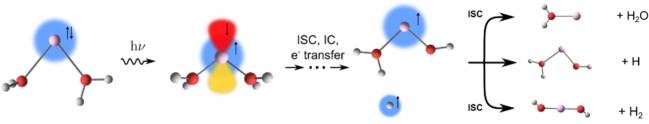

## Introduction

Metal centers with their redox capabilities play a key role in hydrogen evolution, for example, as catalysts in polymer electrolyte membrane electrolyzers^[1]^ or photo‐electrochemical water splitting.^[2]^ Evidence for naturally occurring Al^+^(H_2_O)_*n*_, *n*≤3, was found in mass spectrometry of the ionosphere.^[3]^ Hydrated metal ions in the gas phase are ideal model systems to study the elementary steps of formation of atomic or molecular hydrogen, in particular the relevant charge transfer processes.[^4^, ^5^] In many cases, the unusual +I oxidation state can be obtained in water clusters, sometimes within a limited size regime, resulting in species of the composition M^+^(H_2_O)_*n*_, with, for example, M=Mg, Ca, Al, V, Mn, Fe, Co.[^6^, ^7^, ^8^, ^9^, ^10^, ^11^, ^12^] Some of these systems, like M=Mg, Al, V, are known to eliminate atomic or molecular hydrogen upon heating by room temperature black‐body radiation,[^8^, ^9^, ^13^, ^14^, ^15^] always with very specific dependence on cluster size. These reactions are evident by their characteristic mass changes, leading to metal hydroxide or dihydroxide species.

In the case of Al^+^(H_2_O)_*n*_, H/D exchange experiments with D_2_O indicate that larger clusters are indeed present as HAlOH^+^(H_2_O)_*n*‐1_, but for *n*>40, the exchange ceases,^[16]^ leaving the question open whether these clusters resume the Al^+^(H_2_O)_*n*_ structure or the H/D exchange reaction, which requires efficient proton transfer,^[17]^ becomes hindered. Our recent infrared spectroscopic study confirmed the presence of the HAlOH^+^(H_2_O)_*n*‐1_ structure for *n*=9–14,^[18]^ corroborating the theoretically predicted mechanism of H_2_ formation in these species.[^19^, ^20^] Around *n*=8, the HAlOH^+^(H_2_O)_*n*‐1_ structure is formed.^[19]^ Hydrogen elimination proceeds by recombination of a proton with the hydride. The proton is mobilized by the strongly polarizing character of the Al^III^ center in HAlOH^+^(H_2_O)_*n*‐1_.[^19^, ^20^] Water activation may also take place without hydrogen evolution, as is the case for Mn^+^(H_2_O)_*n*_, which seem to interconvert into HMnOH^+^(H_2_O)_*n*‐1_ in the range of *n*≈8–20,^[11]^ deduced from H/D exchange reactions with D_2_O.

Interestingly, the hydrogen evolution reaction can be triggered by reactions with HCl or HCOOH, which removes the upper limit for its occurrence in Mg^+^(H_2_O)_*n*_, HAlOH^+^(H_2_O)_*n*‐1_ and V^+^(H_2_O)_*n*_.[^14^, ^15^, ^21^] For Zn^+^(H_2_O)_*n*_, uptake of two HCl molecules is required to induce elimination of atomic hydrogen.^[22]^ In reactions with acetonitrile, a hydrogen atom is transferred from Mg^+^(H_2_O)_*n*_, again removing the upper size limit for the formation of MgOH^+^(H_2_O)_*n*‐1_ species,^[23]^ which replicates the chemistry of the hydrated electron.^[24]^ A similar observation was made with CO_2_ and O_2_, which scavenge the hydrated electron present in the hydration shell of Mg^+^(H_2_O)_*n*_,^[25]^ while hydrated transition metal ions exhibit a more complex behavior.^[26]^

Photochemical hydrogen evolution has been reported for a small number of hydrated metal ions in the gas phase to date. The probably best studied system is Mg^+^(H_2_O)_*n*_, *n*≤5, where the unpaired 3s electron of Mg^+^ determines photophysics and photochemistry.[^6^, ^27^, ^28^] The dominant photodissociation product is MgOH^+^(H_2_O)_*m*_, providing clear evidence for photochemical hydrogen evolution. For *n*≥19, the spectra are consistent with the presence of a hydrated electron,[^29^, ^30^] but no photochemical hydrogen evolution takes place. Also Ca^+^(H_2_O)_*n*_, *n*≤6,^[7]^ as well as Co^+^(H_2_O)_*n*_, *n*≤10 and Fe^+^(H_2_O)_*n*_, *n*≤10, undergo photochemical H atom formation in the UV.[^10^, ^31^] Ni^+^(H_2_O) and Co^+^(H_2_O) undergo loss of H_2_O below 18 000 cm^−1^ photon energy.^[32]^

For V^+^(H_2_O)_*n*_, *n*≤12, photochemical hydrogen evolution sets in at photon energies as low as 2.0 eV.^[33]^ Moreover, both atomic and molecular hydrogen are formed, and the branching ratio between the two channels depends sensitively on cluster size and photon energy. For example, in V^+^(H_2_O), H_2_ formation is seen from 2.0 eV while H atom formation sets in only at 2.3 eV and becomes the dominant fragmentation channel above 3.75 eV. H atom loss also dominates over H_2_ elimination for 4≤*n*≤12. For *n*≥15, no photodissociation is observed. This was interpreted as a sign that the clusters have rearranged to HVOH^+^(H_2_O)_*n‐*1_. Since black‐body infrared radiative dissociation (BIRD)^[34]^ induced hydrogen evolution is observed for 9≤*n*≤23,^[9]^ and the HVOH^+^(H_2_O)_*n*‐1_ also contributes to the cluster distribution for *n*≥9,^[33]^ it is obvious that BIRD triggers hydrogen evolution from HVOH^+^(H_2_O)_*n*‐1_ in the electronic ground state while photochemical hydrogen evolution works only with V^+^(H_2_O)_*n*_, which feature suitable electronic transitions. A detailed quantum chemical analysis revealed a dense manifold of quintet and triplet states for V^+^(H_2_O), and photochemical hydrogen formation involved multiple switches of the potential energy surfaces through internal conversion (IC) and intersystem crossing (ISC).^[33]^ This most likely holds true also for larger clusters.

Here, we come back to main group elements and focus on the Al^+^(H_2_O)_*n*_/HAlOH^+^(H_2_O)_*n*‐1_ clusters. Similar to the vanadium case, we also expect a clear spectroscopic signature for the two oxidation states of the metal, which will tell us in which cluster size regime Al^+^(H_2_O)_*n*_ species are actually present. For *n*=1, the Al^+^(H_2_O) structure was experimentally confirmed in ZEKE‐PFI spectroscopy of Al(H_2_O)^[35]^ and IR spectroscopy of Al^+^(H_2_O)Ar.^[36]^ By mass spectrometric analysis of the photofragments, we identify formation of atomic as well as molecular hydrogen. Quantum chemical calculations elucidate the reaction path of photochemical hydrogen evolution from Al^+^(H_2_O)_*n*_. Although our results cannot be directly extended to the condensed phase due to the unusual aluminium oxidation number, they provide mechanistic insight into photochemical hydrogen evolution.

## Experimental Section

The spectroscopic investigations are performed on a modified 4.7 T Bruker Spectrospin CMS47X Fourier‐transform ion cyclotron resonance (FT‐ICR) mass spectrometer^[37]^ coupled with an EKSPLA NT342B tunable optical parametric oscillator (OPO) with a repetition rate of 20 Hz, as described in detail before.[^28^, ^29^, ^30^, ^33^, ^38^] In brief, the clusters are generated by vaporization of an aluminium disk by a frequency doubled Nd:YAG laser.^[39]^ The created plasma containing Al^+^ ions is entrained by a supersonic pulse of He seeded with water vapor, enabling cluster formation. The clusters are then guided to and stored in the ICR cell,^[40]^ covered by a liquid‐nitrogen cooled copper shield to roughly 83 K to minimize the effect of BIRD.^[41]^ As room‐temperature black‐body radiation still partially enters the cell through 6 mm orifices in the front and back trapping plates required for ion and laser entrance, respectively, the equilibrium temperature of clusters will be slightly higher, one can estimate ∼100 K. After mass selection of the ion of interest in the ICR cell, the clusters are irradiated with light provided by the OPO. The amount of dissociation is controlled by a defined number of laser shots fired, usually 5 to 10 shots. After irradiation at a given wavelength, a mass spectrum containing precursor and fragment ions is recorded and the absorption cross‐section can be calculated.^[42]^ This process is repeated for wavelengths of 225–295 nm with step size of 1 nm. The pulse energy is monitored after each mass spectrum to compensate fluctuations of the laser output. Even the cold cryogenic environment of the cooled cell cannot prevent the ions undergoing BIRD dissociation, especially for larger clusters. To correct the influence of fragments caused by BIRD, the fragment intensities of a reference mass spectrum are subtracted from the mass spectra used for calculation of the photodissociation cross‐section. The reference mass spectra are recorded by storing the ions for the same time and at the identical temperature in the ICR cell without laser irradiation.^[33]^

## Computational Methods

All structures are optimized in the ground electronic state at the B3LYP/aug‐cc‐pVDZ level of theory, the minima are verified by frequency calculations and the wave function is always tested for stability. The nature and connections of the transition states (TS) are verified by intrinsic reaction coordinate calculations (IRC) or by distortion along the normal vector of the imaginary frequency in the TS, followed by steepest decent optimization. Electronic transitions are modeled using time‐dependent density functional theory (TD‐DFT), namely TD‐BHandHLYP/aug‐cc‐pVDZ based on its good performance for the investigated 3s–3p excitation in Al^+^, see benchmarking against equation of motion ‐ coupled cluster singles and doubles (EOM‐CCSD) and multireference methods for Al^+^ and Al^+^(H_2_O) in Tables S1 and S2 in the Supporting Information. Natural transition orbitals (NTOs)^[43]^ are calculated at the TD‐BHandHLYP/aug‐cc‐pVDZ level of theory. Photochemical pathways involving conical intersections are modeled using the multi‐reference configuration interaction approach (MRCI), with the active space of 6 electrons in 8 orbitals (6,8), providing ample space for the expected participating 3s and 3p orbitals of Al^+^ in the investigated energy regime. Within complete active space self‐consistent field (CASSCF) calculations, state averaging over four singlet and three triplet states was employed. Spectral shape was modeled using linearized reflection principle,^[44]^ requiring only local information on molecular vibrations in the ground state and forces in each electronically excited state. The Gaussian 16 program^[45]^ is used for geometry optimization, TD‐DFT and EOM‐CCSD calculations while CASSCF and MRCI calculations are performed with Molpro.^[46]^

## Results and Discussion

### Photodissociation of Al^+^(H_2_O)_1‐4_

**Figure 1:**
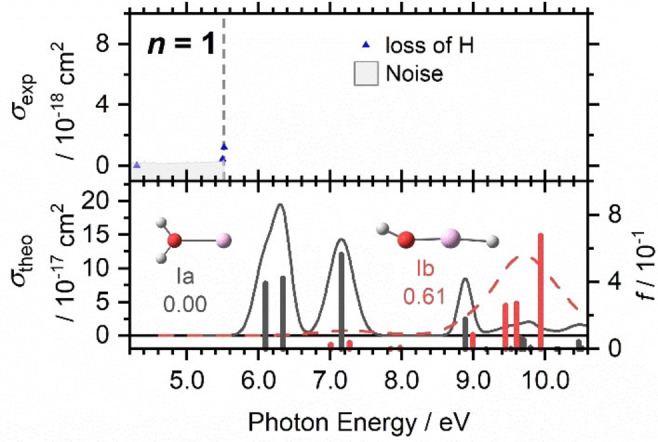
Top: Two data points in the photodissociation cross‐section *σ*_exp_ of Al^+^(H_2_O) at the edge of the experimentally accessible range mark the onset of an absorption, the border of the experimentally accessible energy range is marked by a dashed line. Bottom: The calculated cross‐section *σ*_theo_ plus the corresponding oscillator strengths *f* of transitions in Al^+^(H_2_O) and HAlOH^+^ calculated at the BHandHLYP/aug‐cc‐pVDZ//B3LYP/aug‐cc‐pVDZ level of theory, with spectral width estimated by the linearized reflection principle.

We start our investigation with the aluminium cation hydrated with a single water molecule, shown in Figure 1. Experimentally, loss of H atoms is observed only at the edge of the experimentally accessible range, that is, 5.5 eV. We can provide an upper limit for the experimental cross‐section below 10^−18^ cm^2^, with the more prominent data point reaching 1.2 × 10^−18^ cm^2^. Quantum chemistry predicts transitions of Al^+^(H_2_O) and the inserted HAlOH^+^ only above 6.0 eV, but the rising flank of the first Al^+^(H_2_O) band, which peaks at 6.3 eV, may be responsible for the experimentally observed data points: Simplified spectrum modeling using linearized reflection principle predicts the intensity of 1 × 10^−18^ cm^2^ at 5.6 eV for Al^+^(H_2_O), **Ia**, in good agreement with the experiment. It is illustrative that the excitations of HAlOH^+^ start significantly deeper in the UV in comparison to the cluster with the intact water molecule. The character of the excitations is shown in Figure S1a using NTO orbitals. The energetically lowest lying transition in Al^+^(H_2_O) exhibits a 3s–3p character consistent with the Al^+^ [Ne]3s^2^ electronic configuration. The lowest‐lying excited state features a half‐filled in‐plane Al 3p orbital in the molecular plane. The minimal overlap between the half‐filled 3p orbital and the water lone pairs slightly favors this configuration over the S_2_ state with the half‐filled 3p orbital perpendicular to the molecule plane. The third option, a half‐filled 3p orbital aligned along the Al−O bond, causes an unfavorable overlap with the water valence orbitals, and destabilizes the Al−O bond. However, all three molecular orbitals derived from the Al^+^ [Ne]3s3p configuration have lower excitation energy compared to the atomic ion (7.42 eV),^[47]^ which can be explained by the destabilization of the Al 3s orbital in the Al^+^(H_2_O) ground state due to an anti‐bonding interaction with the water molecule. For HAlOH^+^, the two lowest‐lying transitions correspond to excitation from O 2p lone pair orbitals to an empty antibonding σ* molecular orbital with strong Al 3s contribution, at 7.00 and 7.27 eV. Excitation to a π* MO with dominant Al 3p contribution is also possible, starting at 7.84 eV.

**Figure 2:**
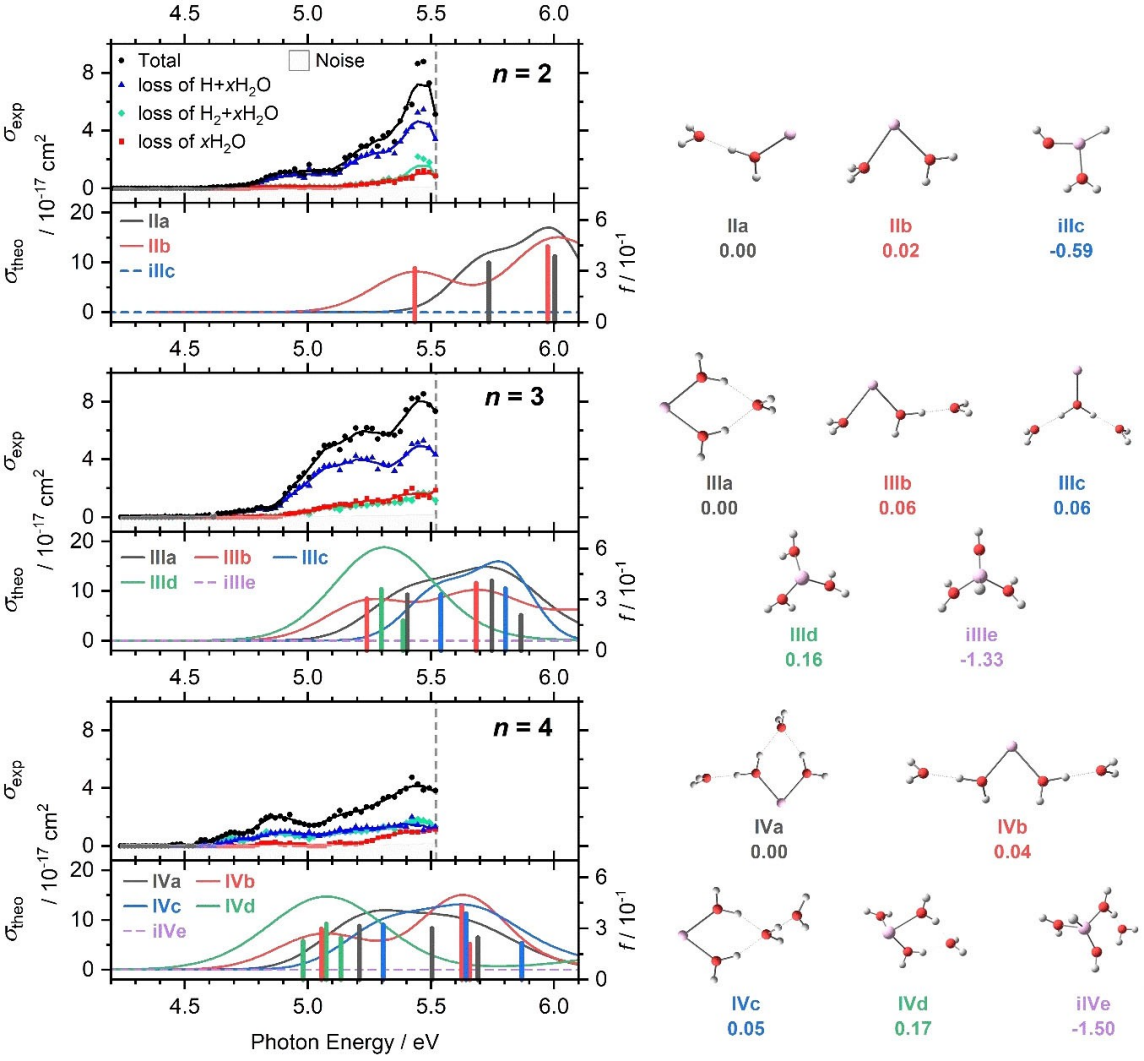
Upper panels: Measured photodissociation cross‐section *σ*_exp_ for Al^+^(H_2_O)_*n*_, *n*=2–4, along with a five‐point running average, broken down into the chemically distinct channels, loss of atomic or molecular hydrogen and water evaporation. The border of the experimentally accessible energy range is marked by a dashed line. Lower panels: Theoretical cross‐section *σ*_theo_ and the corresponding oscillator strengths *f* calculated at the BHandHLYP/aug‐cc‐pVDZ//B3LYP/aug‐cc‐pVDZ level of theory, with spectral width estimated through linearized reflection principle. Right: Isomers optimized at the B3LYP/aug‐cc‐pVDZ level; relative energy is given in eV; inserted isomers are labeled by “i”.

Intense absorptions are observed for photodissociation of Al^+^(H_2_O)_*n*_, *n*=2–4, as shown in Figure 2. Photodissociation starts at about 4.5–4.6 eV, reaching cross‐sections in the high 10^−17^ cm^2^ range for *n*=2, 3, with somewhat smaller values for *n*=4. As four stages of nonlinear optics are used to generate tunable laser light in this wavelength region, the pulse energy strongly fluctuates, which leads to the relatively strong scattering of the data points. We therefore provide a running average across five data points to guide the eye. Three fragmentation channels are observed: loss of water molecules, *x*H_2_O (red), elimination of atomic hydrogen, H+*x*H_2_O (blue), and elimination of molecular hydrogen, H_2_+*x*H_2_O (green), the latter two accompanied by loss of water. For *n*=2 or 3, loss of a hydrogen radical is the most prominent channel while the formation of a hydrogen molecule becomes competitive for *n*=4. Pure water loss (red) overall plays a minor role, but exhibits a steep rise at higher photon energies for *n*=4.

Quantum chemical calculations reveal that many almost isoenergetic isomers exist, with water molecules in different solvation shells (Figure 2), as reported before by Watanabe and Iwata.^[48]^ The lowest‐lying structure of Al^+^(H_2_O)_*n*_ contains one, two and two water molecules in the first solvation shell for *n*=2, 3, 4. In other words, adding water to the second solvation shell with favorable hydrogen bonds is preferred to Al^+^ core with three coordinated water molecules. The inserted structure, HAlOH^+^(H_2_O)_*n*‐1_, is energetically favored for *n*≥2, and additional hydration leads to increased stabilization. However, CID experiments by Armentrout and co‐workers^[49]^ are consistent with an Al^+^(H_2_O)_*n*_ structure for *n*≤4.

Excited state calculations show that HAlOH^+^(H_2_O)_*n*‐1_ stays transparent in the investigated energy range, thus the observed photodissociation is exclusively due to absorptions of Al^+^(H_2_O)_*n*_, even though HAlOH^+^(H_2_O)_*n*‐1_ is thermochemically favored. Still, this does not rule out that a mixture of Al^+^(H_2_O)_*n*_ and HAlOH^+^(H_2_O)_*n*‐1_ is present in the experiment.

The character of the excitations is investigated Figure S1b for *n*=2. The second coordinated water molecule in **IIb** isomer destabilizes the ground state more than the three lowest‐lying excited states, causing these transitions to shift down to 5.43, 5.97, and 6.26 eV. For HAlOH^+^(H_2_O), **iIIc**, the lowest lying excitations are blue‐shifted to 7.80 and 7.95 eV.

The calculations provide good agreement with the experimental position and intensity of the bands, although the linearized reflection principle is not able to capture the spectral onset for *n*=2. Since the redshift of the 3s–3p excitations strongly depends on the position and orientation of the water molecules interacting with the Al^+^ 3s and 3p orbitals, the excitations into the lowest excited state differ by up to 0.3 eV between low‐lying isomers. Therefore, very broad bands can be expected in the experiment due to the presence of several isomers at experimental temperatures, consistent with the experimental spectra in Figure 2. The absorption spectrum calculations confirm that Al^+^(H_2_O)_*n*_ is predominantly present in the experiment. However, the calculated cross‐sections are slightly higher than the experimental ones, up to a factor of ∼3; this may be explained, apart from the error of the computational model, by the presence of transparent HAlOH^+^(H_2_O)_*n*‐1_ in the experimental mixture. This structure has been reported for *n*=2 in earlier infrared photodissociation experiments by Inokuchi et al.^[36]^ Here, the decreasing cross‐section in the experiment for *n*=4 could indicate that the transparent HAlOH^+^(H_2_O)_*n*‐1_ becomes more prominent with increasing cluster size.

### Photochemical modeling

**Figure 3:**
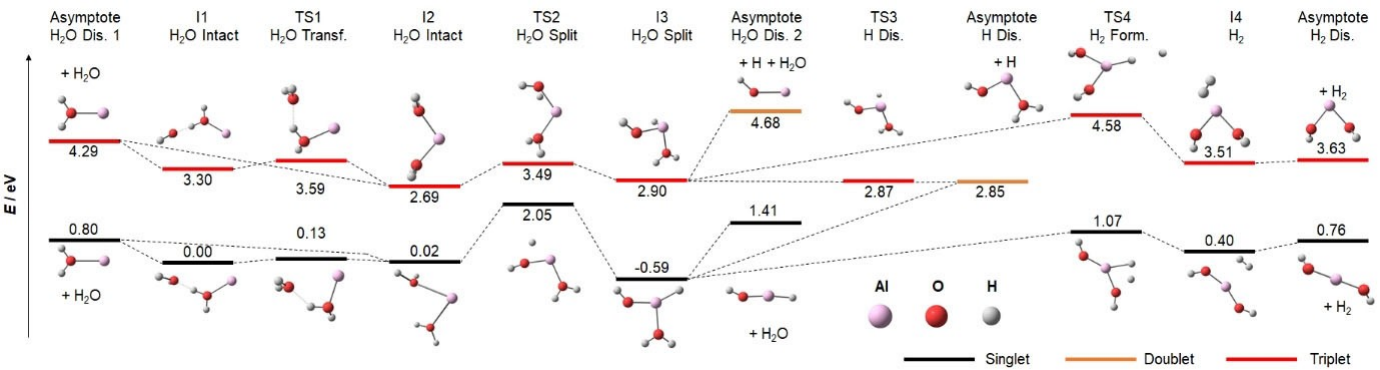
Potential‐energy surface for the decomposition of Al^+^(H_2_O)_2_ towards water loss, atomic hydrogen loss and molecular hydrogen formation in the singlet ground state and lowest‐lying triplet state calculated at the B3LYP/aug‐cc‐pVDZ level of theory. For the second H_2_O triplet asymptote, HAlOH^+^ dissociates spontaneously to AlOH^+^+H.

We start our investigation of the photochemistry by modeling the observed decomposition involving the loss of H, H_2_ or H_2_O on the singlet ground state and lowest‐lying triplet potential energy surfaces (PES) for *n*=2, see Figure 3. The *n*=1 PES has been studied before.^[50]^ The minima I1 and I2 with one and two water molecules in the first solvation shell, respectively, are almost isoenergetic, connected by TS1 at 0.13 eV in the singlet state. Water loss from both minima is relatively simple at a cost of 0.80 eV. The insertion of the aluminium center into an O−H bond is energetically most facile when both water molecules are coordinated to Al^+^. However, the exothermic product I3, HAlOH^+^(H_2_O) at −0.59 eV, is well separated from I2 by TS2 at 2.05 eV. From I3, H atom loss at 2.85 eV lies well above water loss at 1.41 eV. H_2_ formation, on the other hand, can now readily proceed via TS4 at 1.07 eV, leading to Al(OH)_2_^+^+H_2_ at 0.76 eV. The formation of hydrated aluminium oxide is not competitive, requiring at least 3.64 eV without considering transition states (not shown in Figure 3). Looking at the overall energetics, H_2_O loss is expected for Al^+^(H_2_O)_2_ species I1 or I2 while H_2_ loss with aluminium dihydroxide formation must be expected for HAlOH^+^(H_2_O) (I3), with both pathways well separated.

On the triplet PES, H_2_O evaporation with 4.29 eV lies significantly above the barrier for insertion, TS2 at 3.49 eV. From I3, the hydrogen atom is readily lost along a downhill path via TS3, which is placed below I3 by zero‐point correction. Molecular hydrogen formation via TS4, on the other hand, is prohibited, facing a barrier with TS4 at 4.58 eV. Because H_2_O loss is preferred on the singlet surface and H atom loss seems inevitable in the lowest‐lying triplet state, the experimentally observed formation of molecular hydrogen from Al^+^(H_2_O)_2_ is clearly of photochemical origin. However, photochemistry may also provide pathways for H and H_2_O loss that differ from this analysis of the singlet ground state and the lowest lying triplet state PESs.

**Figure 4:**
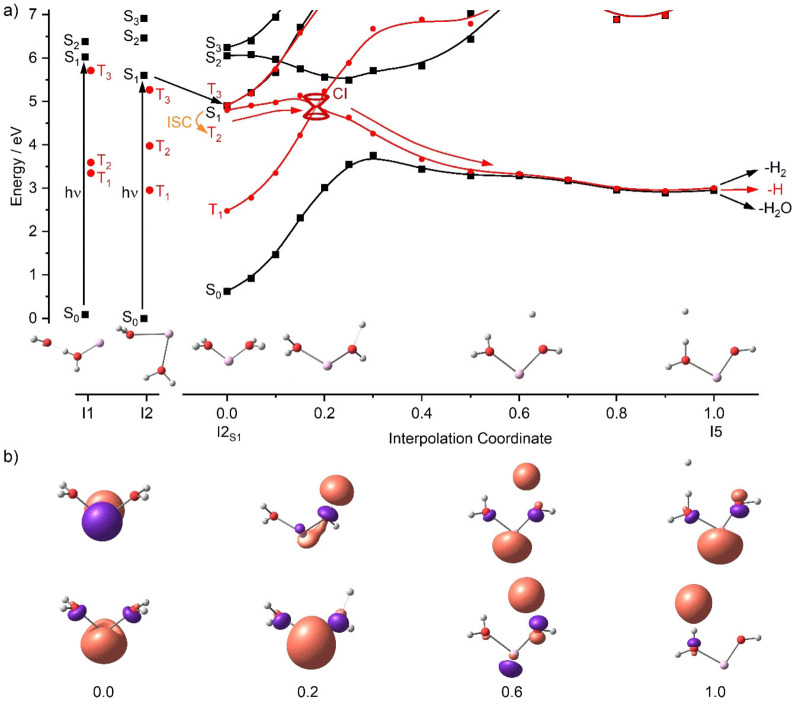
a) Calculated excitation energies in the Franck Condon point for both isomers of Al^+^(H_2_O)_2_, I1 and I2, followed by relaxation into excited state minimum I2_S1_ of S_1_, which defines position 0.0 of the interpolation coordinate, 1.0 corresponds to T_1_/S_0_ minimum I5. I1, I2 and I5 are optimized at the B3LYP/aug‐cc‐pVDZ level of theory; I2_S1_ is optimized with BHandHLYP/aug‐cc‐pVDZ. All data points represent single‐point energies calculated at the MRCI(6,8)/aug‐cc‐pVDZ level without zero‐point correction. b) For the 0.0 point of the interpolation coordinate, natural transition orbitals for S_1_ at BHandHLYP/aug‐cc‐pVDZ are shown; for 0.2 and above, the two highest‐lying α‐spin orbitals in T_1_, are depicted as calculated at B3LYP/aug‐cc‐pVDZ.

We continue our investigation by an analysis of the first three excited states in singlet and triplet spin multiplicity, S_1_–S_3_ and T_1_–T_3_, on the MRCI(6,8)/aug‐cc‐pVDZ//BHandHLYP/aug‐cc‐pVDZ level of theory, Figure 4. As there are two almost isoenergetic isomers in the ground state, we calculate the excitation energy in two Franck‐Condon points of I1 and I2, corresponding to isomers **IIa** and **IIb**, respectively. The triplet states T_1_–T_3_ as well as S_1_ lie energetically lower at I2 than I1, suggesting that the system may relax from the I1 towards the I2 structure upon photoexcitation at I1. Indeed, both minima are interconnected by a transition state TS_S1_ with energy by 0.12 eV higher than the S_1_ excitation energy in I1, that is, at 5.86 eV, and can be thus surpassed if enough thermal energy is available (BHandHLYP/aug‐cc‐pVDZ values; Figure S2).

For I1, the minimum in the first excited singlet state does not differ considerably from the Franck Condon point, with the energy lowered by only 0.22 eV through optimization in the S_1_ state (Figure S2). I2, however, undergoes a significant structural change in S_1_, leading towards the local minimum I2_S1_ depicted in Figure 4a, with the former in‐plane water molecule rotating out of plane to afford C_2v_ symmetry of the cluster. This local S_1_ minimum lies 0.74 eV below S_1_ in I2 structure. However, S_1_ and S_0_ are still well separated by more than 4 eV, and no conical intersections (CI) between these two states seem to be accessible in the vicinity. Decomposition on the S_1_ PES can be ruled out on energetic grounds. A multiphoton process seems unlikely, in view of the high intensity observed in the experimental spectra in good agreement with the calculated intensities. Fluorescence from the I2_S1_ minimum would bring the system to the S_0_ state in I2_S1_ geometry, with up to 1.6 eV of relaxation energy available for water evaporation (excitation energy of 5.9 and 5.6 eV in I1 and I2, respectively, minus the S_0_–S_1_ gap in I2_S1_ of 4.3 eV at the MRCI(6,8)/aug‐cc‐pVDZ level; for BHandHLYP/aug‐cc‐pVDZ, the relaxation energy reaches up to 1.5 eV). However, this energy is not sufficient for hydrogen atom or hydrogen molecule formation. Therefore, fluorescence can be ruled out as the dominant relaxation pathway since preferred loss of atomic hydrogen is experimentally observed.

The conclusion of Figure 3 was that atomic hydrogen formation is straightforward in the triplet state T_1_, which is only accessible after intersystem crossing (ISC). Interestingly, T_2_ and T_3_ are almost isoenergetic with S_1_ in the I2_S1_ minimum, interpolation coordinate 0.0 in Figure 4a, making ISC possible. The triplet states T_1_–T_3_ closely correspond to the S_1_–S_3_ states with one unpaired electron each in the 3s and 3p orbital of Al, and they lie energetically below their singlet counterparts.

An extensive search of local minima on various excited state potential energy surfaces revealed an interesting biradical structure I5, with a hydrogen radical loosely coordinated to a hydrogen atom of the remaining water molecule, which with 2.80 eV even lies below the HAlOH^+^(H_2_O) minimum I3 in the triplet state. However, I5 will only play a role in the photochemical reaction if it can be reached from I2_S1_. We therefore performed a linear interpolation between I5 and I2_S1_ and calculated S_0_–S_3_ and T_1_–T_3_ single‐point energies along the interpolation coordinate on the MRCI(6,8)/aug‐cc‐pVDZ level of theory. The resulting curves, Figure 4a, indicate that S_1_ indeed undergoes ISC to T_2_, and the available energy easily suffices to reach the CI between T_2_ and T_1_. Passing through this CI, T_2_ changes to T_1_ along the interpolation coordinate. The system further relaxes to I5, along a path where T_1_ and S_0_ become degenerate, indicating that the hydrogen radical and the unpaired Al 3s electron do not interact significantly.

Natural transition orbitals in S_1_ and the two highest‐lying alpha‐spin orbitals in T_1_, which largely correspond to the unpaired electrons, illustrate how the reaction proceeds mechanistically along the interpolation coordinate (Figure 4b). Around the CI, interpolation coordinate 0.2, the Al 3p electron is transferred to a water ligand, occupying an antibonding σ* orbital. This weakens the O−H bond and transforms the water molecule into the OH^−^⋅⋅⋅H structure, which fully evolves at interpolation coordinate 0.6. Further relaxation towards the I5 minimum transfers the H atom to the intact water ligand, with which it interacts very weakly. This picture is mirrored in the partial charges and spin densities along the interpolation coordinate (Figure S3). At the IC, interpolation coordinate 0.2, spin density on Al is still ∼1.5, but the dissociating H atom has already acquired spin density of ∼0.5. The spin density of almost exactly 1 at Al and H for interpolation coordinate ≥0.4 indicates that the H atom is fully dissociated, and zero spin density at OH is consistent with a fully developed OH^−^ moiety. The values of the calculated partial charges indicate a similar trend. For technical reasons, CHELPG charge and spin density can only be calculated after the CI, when T_1_ is the relevant triplet state for the reaction. In the T_2_ region of the interpolation path, we have to resort to less reliable Mulliken charges. These extrapolate the picture to the I2_S1_ minimum, and are consistent with the transfer of one electron from the metal center to the water ligand in the 0.0–0.4 region of the interpolation coordinate (Figure S3).

**Figure 5:**
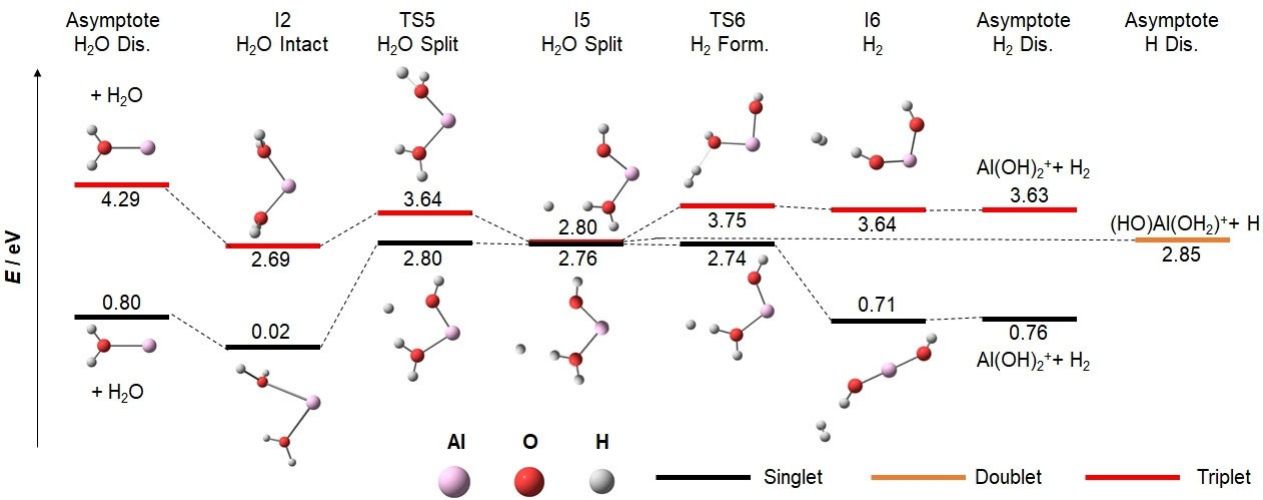
Potential energy surface for the decomposition pathways in singlet and triplet spin multiplicity after photochemical relaxation of Al^+^(H_2_O)_2_ to the I5 minimum. Calculated on the B3LYP/aug‐cc‐pVDZ level of theory.

The analysis in Figure 4 indicates that I5 is reached efficiently after photoexcitation. To fully understand molecular and atomic hydrogen formation in competition with water loss, we performed additional analysis of the reaction PES, shown in Figure 5. From the I5 minimum, a hydrogen radical can readily dissociate as the preferred path in the triplet state while water loss via TS5 or H_2_ formation via TS6 at 3.64 and 3.75 eV, respectively, face appreciable barriers. The energy is available, but statistics disfavors the passage of high‐lying tight transition states. However, if we consider spin crossing back to the singlet surface, both transition states are significantly lowered, becoming almost isoenergetic with the I5 minimum. Therefore, H_2_O as well as H_2_ elimination will take place after ISC to the singlet surface while atomic hydrogen formation is preferred in the triplet state.

**Scheme 1:**
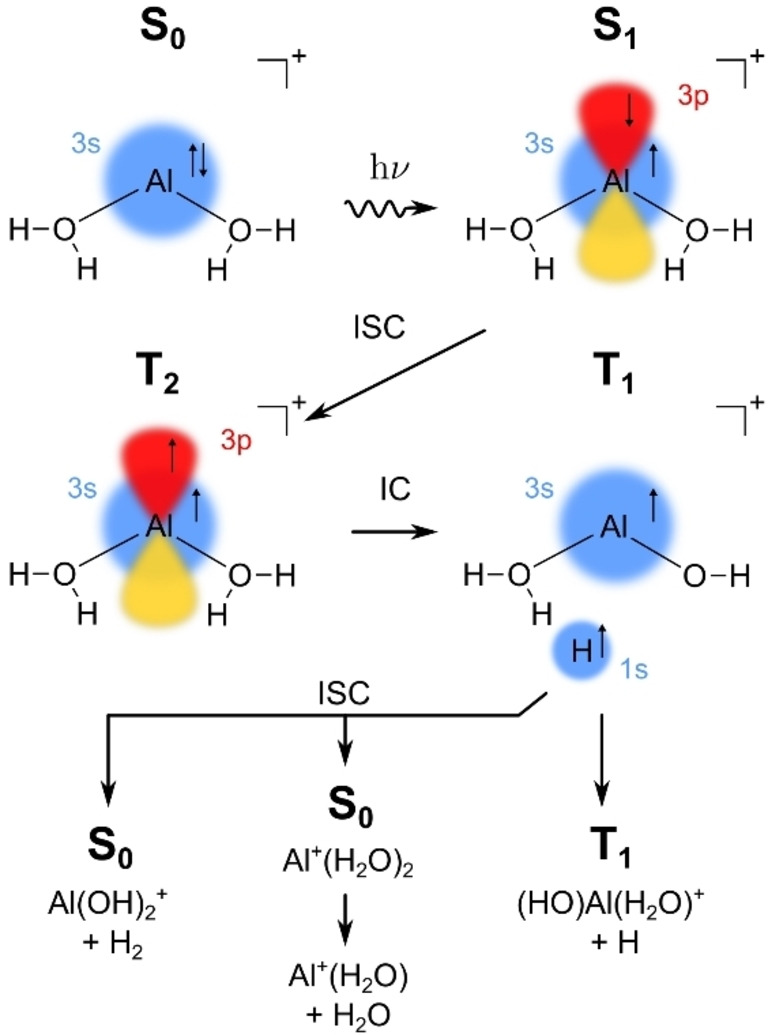
Suggested reaction pathway after excitation of Al^+^(H_2_O)_2_ into the S_1_ state, including intersystem crossing (ISC) and internal conversion (IC).

The analysis of potential photochemical reaction paths in Figures 3–5 is summarized in Scheme 1. It indicates that after photoexcitation, the molecule relaxes to the I2_S1_ minimum and waits to undergo ISC to T_2_. Passing through a CI to T_1_ and further relaxation leads to I5, in which an O−H bond is broken and a hydrogen atom is loosely attached to the intact water molecule. The loosely bound hydrogen radical either evaporates directly (the dominant pathway in the experiment) or recombines with OH or attacks H_2_O on the singlet ground state surface to lose H_2_O or H_2_ as observed in the experiment.

Because the experimental product branching ratio is very similar for *n*=3, one may speculate that the photochemical mechanism is analogous, despite the increased absorption intensity at lower photon energies. For *n*=4, the situation changes significantly, with equal contributions of H and H_2_ loss while water loss becomes significant only at photon energies above 5.2 eV. With the redshift of the transitions and the increased conformational flexibility of the larger cluster, more photochemical reaction pathways will become available. Photodynamics is sensitive to the excited state reached after excitation, and the intriguing behavior of the *n*=4 branching ratio indeed suggests that an S_1_/S_0_ CI to the ground state may become accessible at higher photon energies, but this is only a speculation.

The photochemical decomposition can be expected to be extremely complicated in detail and heavily dependent on the cluster structure, exact energetic location of the available ISCs, ICs and transition states. In any case, a more quantitative picture would require molecular photodynamics employing multi‐reference methods including spin flips,^[51]^ which is beyond the scope of the present work.

### Photodissociation of Al^+^(H_2_O)_n,_ n>4

**Figure 6:**
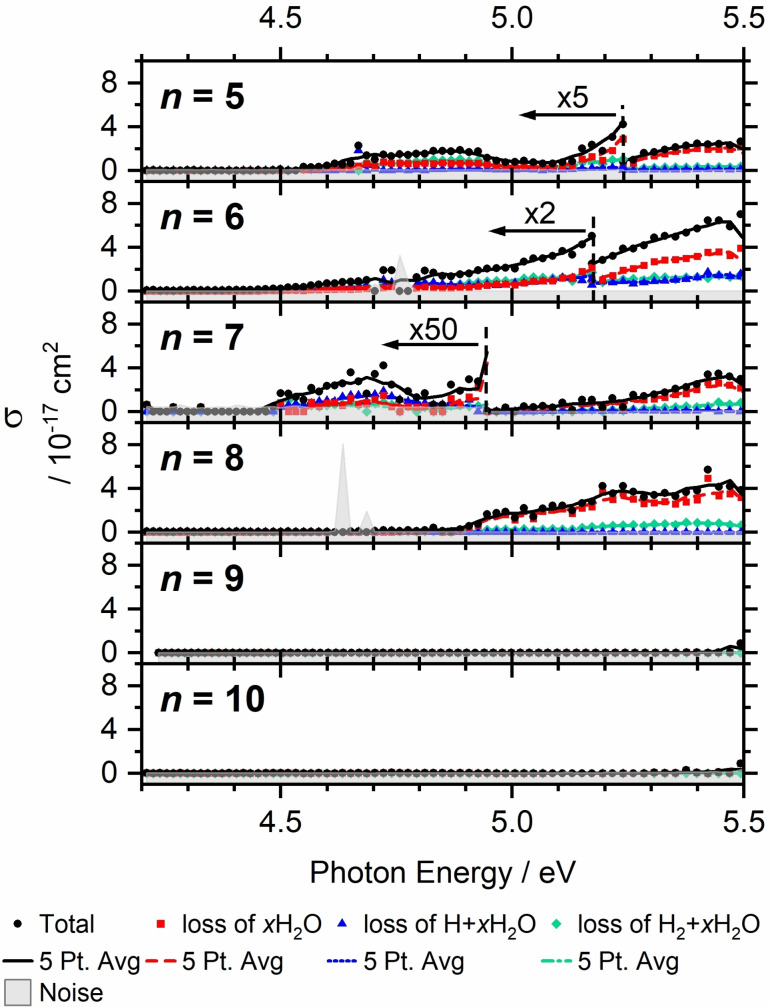
Photodissociation cross‐section σ of Al^+^(H_2_O)_*n*_, *n*=5–10. Weak transitions are scaled as indicated for better visibility.

Photodissociation of larger cluster with *n*>4 was only studied experimentally for cluster sizes *n*=5–10 (Figure 6). For *n*=5–8, absorptions are observed from 4.5–5.5 eV, similar to *n*=4, with the cross‐section increasing with photon energy, reaching values in the 10^−17^–10^−18^ cm^2^ region. However, the cross‐section strongly depends on cluster size. For clusters with *n*=9, 10, some evidence for an absorption is obtained only at the high energy end of the available photon energies, albeit at a relatively high noise level.

It is well established that Al^+^(H_2_O)_*n*_ rearranges to HAlOH^+^(H_2_O)_*n*‐1_, and we observe the Al−H stretching mode in our infrared spectra in traces for *n*=4, with quickly increasing intensity for larger clusters.^[18]^ The inserted structure HAlOH^+^(H_2_O)_*n*‐1_ does not absorb UV light with *λ*≥225 nm for *n*=2–4, and hydration does not seem to lead to an appreciable redshift. We therefore assign the size‐dependence of the absorption cross‐section and the disappearance of the absorption to the presence of the HAlOH^+^(H_2_O)_*n*‐1_ structure. As the intensities fluctuate, we conclude that the conversion of Al^+^(H_2_O)_*n*_ into HAlOH^+^(H_2_O)_*n*‐1_ due to its exothermicity[^19^, ^20^] is followed by evaporation of 4 or 5 water molecules and is also size dependent. It starts around *n*=8 or 9, assuming that at least four water molecules evaporate. At *T*≈100 K, interconversion of Al^+^(H_2_O)_*n*_ is obviously slow for *n*≤8 since these species exhibit intense UV absorptions on the timescale of the experiment, which is several seconds. However, *n*=9 is almost completely converted, indicating that this cluster size readily interconverts under the experimental conditions, and only a small fraction of Al^+^(H_2_O)_*n*_ remains.

The absence of further redshift for *n*>4 is assigned to an asymmetric solvation of the Al^+^ core,^[4]^ with water molecules added to higher solvation shells instead of binding directly to the metal center. This suggests that Al^+^ remains doubly coordinated for *n*≤8.

For the photochemical decomposition of *n*=5–8, water loss becomes the dominant decomposition channel towards higher photon energies. Hydrogen molecule formation is observed in all four cases. H atom loss, however, is suppressed for *n*=5 and *n*=8 while it occurs at competitive intensities for *n*=6 or 7. For *n*=7, it is even the dominant photochemical reaction path below 4.8 eV, but disappears around 5.0 eV. As discussed above, increasing solvation statistically favors water evaporation. Water loss can occur, for example, after excitation followed by fluorescence. This scenario is supported by the experimental trend towards higher photon energies where only one or two water molecules are lost while the photon energy is sufficient for evaporation of almost the complete hydration shell. Accessibility of IC or ISC depends sensitively on cluster structure, and a single water molecule might lead to significant changes in photochemical behavior. One factor in the suppression of the H atom loss channel is the integration of the first shell water molecules into the hydrogen‐bonded network, which reduces their conformational flexibility. However, this also applies to the H_2_ elimination pathways, which are still active for *n*=8. Unfortunately, quantum chemical analysis of the photochemistry becomes difficult with increasing cluster size, with each water molecule adding nine dimensions to the potential energy surface of the cluster.

## Conclusion

Photodissociation spectroscopy confirms the transition from Al^+^(H_2_O)_*n*_ to HAlOH^+^(H_2_O)_*n*‐1_ in hydrated aluminium cations with increasing cluster size, and for *n*=9 or 10, only the inserted structure is present, as evidenced by the lack of measurable absorption cross‐section for wavelengths above 225 nm. While black‐body infrared radiative dissociation (BIRD) triggers molecular hydrogen formation from HAlOH^+^(H_2_O)_*n*‐1_, photochemical reaction pathways lead to the evolution of atomic as well as molecular hydrogen from Al^+^(H_2_O)_*n*_. For *n*≤4, the formation of H atoms is preferred over molecular hydrogen elimination. Quantum chemical analysis reveals that a singlet–triplet intersystem crossing is required to explain all observed photochemical reaction products. For *n*=2, the Al 3p electron in the S_1_ state is transferred to a σ* orbital of a water ligand; this weakens the O−H bond and ultimately leads to formation of atomic hydrogen and a hydroxide ligand. Before its final release, the roaming H atom might abstract hydrogen from the intact water ligand, thus explaining H_2_ formation, or recombine with the hydroxide with concomitant electron transfer back to the metal center, essentially reversing the reaction. The accumulated relaxation energy in the cluster triggers water evaporation in the electronic ground state. Photochemical hydrogen evolution in hydrated aluminium is thus fundamentally different from the known ground‐state reaction induced by BIRD, and does not involve a HAlOH^+^(H_2_O)_*n*‐1_ hydride‐hydroxide structure.

## Conflict of interest

The authors declare no conflict of interest.

## Supporting information

As a service to our authors and readers, this journal provides supporting information supplied by the authors. Such materials are peer reviewed and may be re‐organized for online delivery, but are not copy‐edited or typeset. Technical support issues arising from supporting information (other than missing files) should be addressed to the authors.

Supporting Information
