# Supplementary material for: Photochemical Hydrogen Evolution at Metal Centers Probed with Hydrated Aluminium Cations, Al+(H2O) n , n=1–10
Source: Chemistry. 2021 Nov 5;27(66):16367–76. doi: 10.1002/chem.202103289 (PMC9298212; doi:10.1002/chem.202103289)
Supplement: Supplementary file 1 — Supporting Information [file CHEM-27-16367-s001.pdf]

Chemistry–A European Journal

Supporting Information

Photochemical Hydrogen Evolution at Metal Centers Probed with Hydrated Aluminium Cations, $Al^+(H_2O)_n$, $n = 1–10$

Jakob Heller, Tobias F. Pascher, Christian van der Linde, Milan Ončák,* and Martin K. Beyer*

Table S1. Electronic configuration, degeneracy and excitations energies in the Al^+ ion calculated at various levels of theory (in eV), in comparison to experimental values.

conf.	deg.	EOM-CCSD/ 6-31+g*	EOM-CCSD/ aug-cc-pVDZ	EOM-CCSD aug-cc-pVTZ	CASSCF(2,13)/ aug-cc-pVDZ	MRCI(2,13)/ aug-cc-pVDZ	BhandHLYP/ aug-cc-pVDZ	exp.[a)]
3s3p	3	7.67	7.50	7.41	7.58	7.50	7.49	7.42
$3p^2$	5	11.63	10.53	10.41	10.42	10.51	11.94	10.60
3s4s	1	11.69	11.69	11.69	11.71	11.68	11.43	11.82
3s4p	3	13.17	13.30	13.24	13.31	13.29	12.79	13.26

a) NIST database: Kramida, A., Ralchenko, Yu., Reader, J., and NIST ASD Team (2020). NIST Atomic Spectra Database (ver. 5.8), [Online]. Available: https://physics.nist.gov/asd [2021, August 30]. National Institute of Standards and Technology, Gaithersburg, MD. DOI: https://doi.org/10.18434/T4W30F

Table S2. Excitation energies of three 3s-3p transitions in the $Al^+(H_2O)$ ion calculated at various levels of theory (in eV) with the structure optimized at the B3LYP/aug-cc-pVDZ level of theory.

state	EOM-CCSD/6-31+g*	EOM-CCSD/aug-cc-pVDZ	EOM-CCSD/aug-cc-pVTZ	BhandHLYP/aug-cc-pVDZ
S_1	6.23	6.06	6.00	6.09
S_2	6.55	6.34	6.27	6.34
S_3	7.53	7.34	7.29	7.15

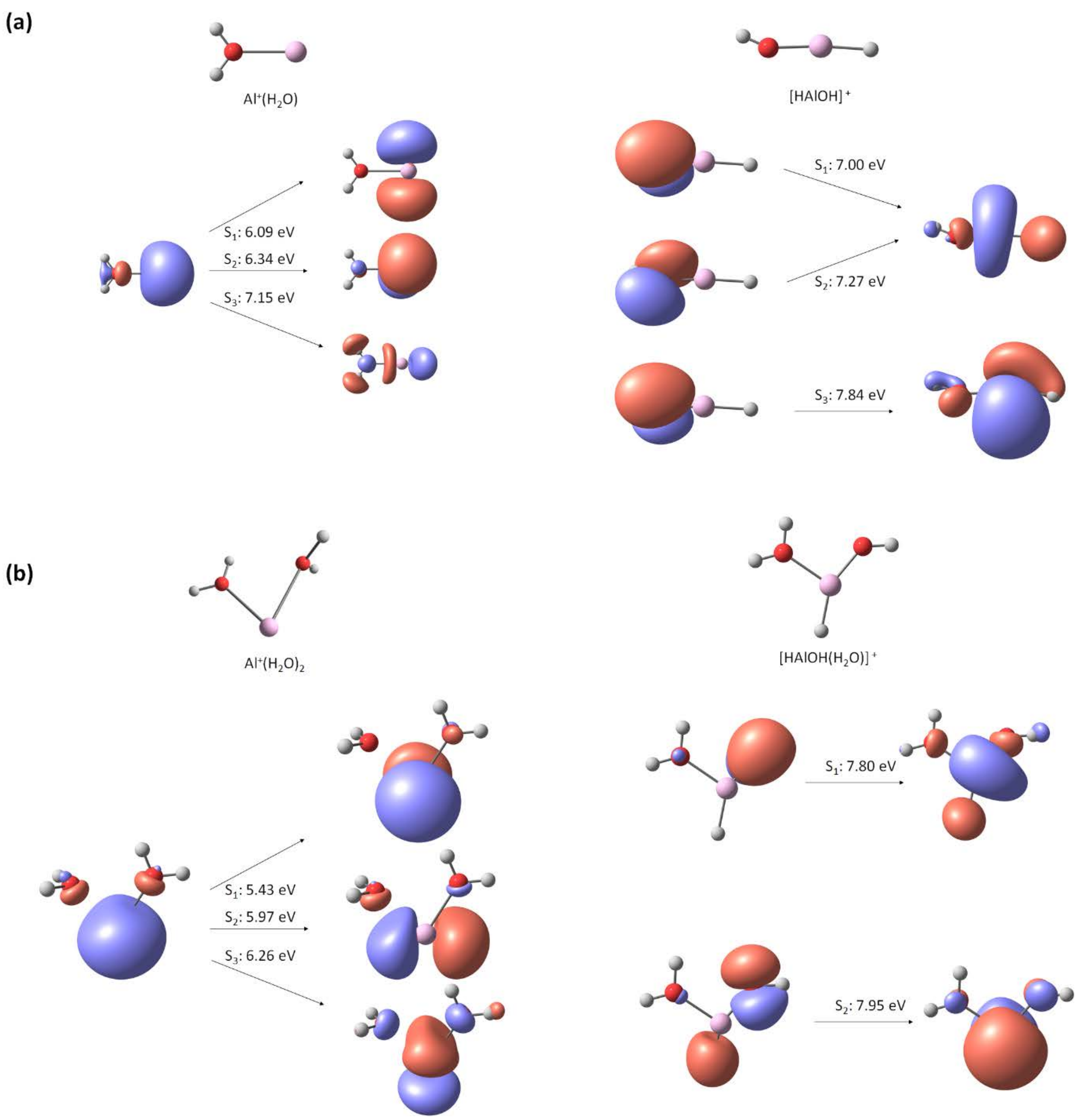

Figure S1. Character of the energetically lowest lying excitations of the a) $Al^+(H_2O)$ along with its $[HAlOH]^+$ counterpart and b) $Al^+(H_2O)_2$ along with its $[HAlOH(H_2O)]^+$ counterpart. Depicted transition energies and NTOs calculated at the BHandHLYP/aug-cc-pVDZ//B3LYP/aug-cc-pVDZ level of theory.

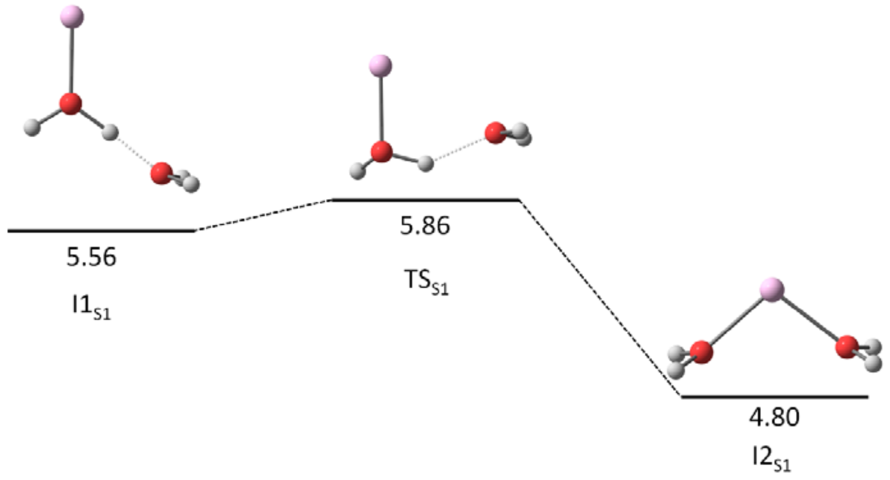

Figure S2. Two minima obtained by optimizing I1 and I2 structures in the S_1 state, $I1_{S1}$ and $I2_{S1}$, and the connecting transition state, TS_{S1}. Calculated at the BHandHLYP/aug-cc-pVDZ level of theory, relative energy is given with respect to the I1 minimum optimized at the B3LYP/aug-cc-pVDZ level. Zero point energy is included in the reported values.

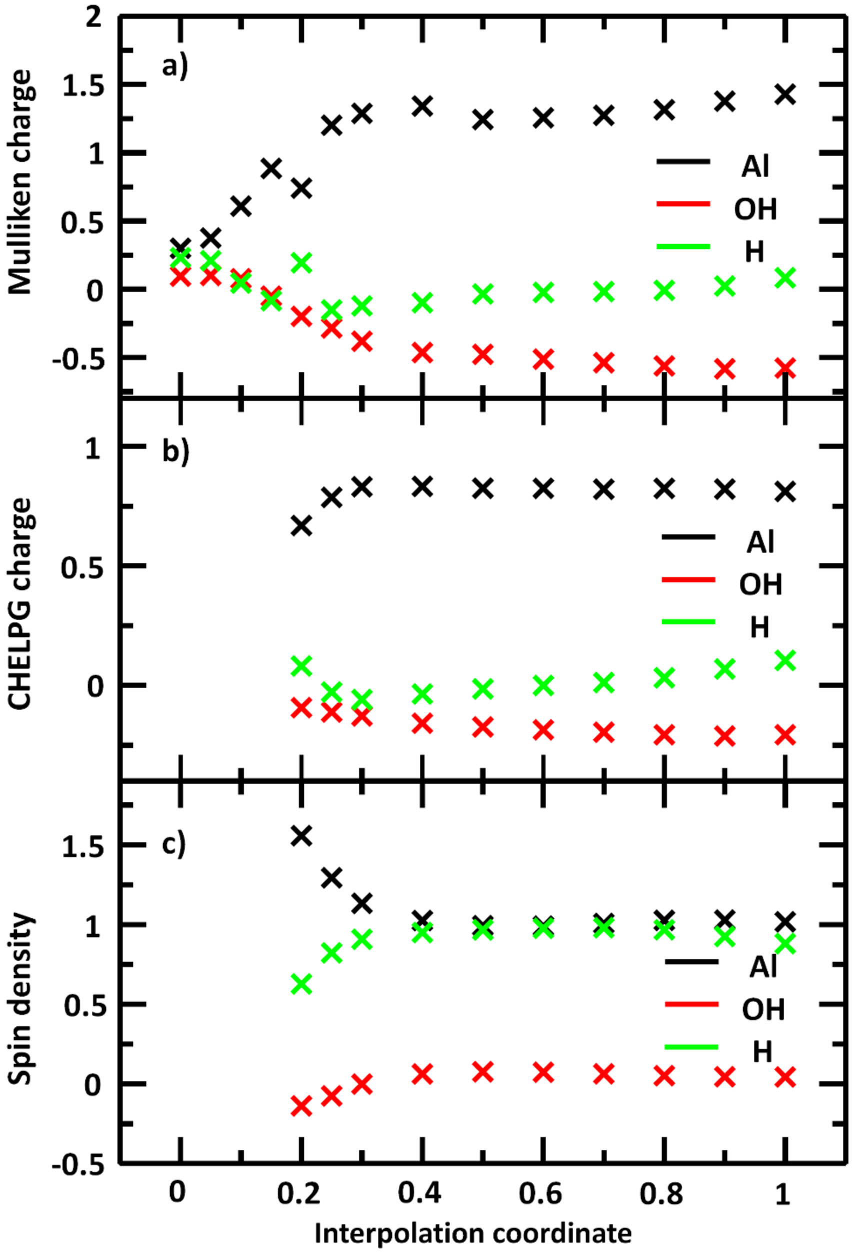

Figure S3. Various properties of Al, OH and H groups in the T_2 and T_1 states along the interpolation coordinate before and after the CI, respectively, shown in Figure 4. The conical intersection is located at point ~0.2. a) Mulliken charges as calculated at the CASSCF(6,8)/aug-cc-pVDZ level. b) CHELPG charges as calculated at the B3LYP/aug-cc-pVDZ level. c) Spin density as calculated at the B3LYP/aug-cc-pVDZ level.

Cartesian coordinates of optimized ions and molecules (in Å, calculated at the B3LYP/aug-cc-pVDZ level) along with electronic energies (in Hartree) including zero-point correction and the corresponding spin multiplicity *M* if it differs from the lowest one.

```
Ia
E = -318.625537
Al -0.000000 -0.000000 0.980754
O 0.000000 0.000000 -1.159061
H 0.000000 0.781657 -1.738654
H -0.000000 -0.781657 -1.738654

Ib
E = -318.603068
o 0.011964 1.023150 0.000000
al 0.011964 -0.608618 -0.000000
h -0.432973 1.874676 -0.000000
h 0.181733 -2.147839 0.000000

IIa
E = -395.078341
O 0.103397 0.719843 -0.000020
Al 1.802961 -0.347135 0.000004
O -2.232121 -0.316120 -0.000010
H -0.848066 0.344429 -0.000503
H 0.049020 1.689114 0.000282
H -2.805115 -0.375262 -0.776644
H -2.804532 -0.375312 0.777051

IIb
E = -395.077594
O 1.456801 -0.527367 -0.000112
Al -0.162942 1.067665 0.000102
O -1.299973 -0.738967 -0.000075
H 2.028934 -0.652840 0.775002
H 2.032402 -0.647560 -0.773489
H -2.267965 -0.812086 0.000662
H -0.929744 -1.636489 -0.001999

IIc
E = -395.099955
O -1.393755 -0.261865 -0.000000
Al 0.000000 0.676490 0.000000
O 1.215067 -0.779235 0.000000
H -2.330797 -0.046877 -0.000000
H 0.705459 2.056863 0.000000
H 2.185742 -0.786082 0.000000
H 0.869094 -1.689478 -0.000000

IIIa
E = -471.528413
O -0.134432 -1.349338 -0.000246
Al -1.762140 0.000309 0.000188
O -0.133599 1.349233 -0.000300
H 0.824304 -1.128804 -0.000269
H -0.241872 -2.311579 -0.000337
H -0.240204 2.311568 0.000403
H 0.824963 1.128011 0.000105
O 2.270965 -0.000177 0.000029
H 2.857456 -0.001489 0.771311
H 2.859701 0.000525 -0.769534

IIIb
E = -471.526320
O -0.421591 -0.412380 0.000390
Al 0.919066 1.093275 0.000048
O 2.301020 -0.756784 -0.000204
H -0.173948 -1.349098 0.000500
H -1.427004 -0.328745 -0.000048
H 2.861931 -0.932569 -0.772922
H 2.862024 -0.932864 0.772368
O -2.995728 -0.127399 -0.000166
H -3.570458 -0.148741 0.775971
H -3.570021 -0.148061 -0.776645

IIIc
E = -471.526239
O 0.000332 0.117949 -0.000042
Al -0.001302 2.057546 0.000009
O -2.211765 -1.257234 0.000053
O 2.213418 -1.254651 0.000070
H -0.824026 -0.460048 0.000307
H 0.825189 -0.459629 0.000085
H -2.614466 -1.670070 -0.775199
H -2.617703 -1.669435 0.773946
H 2.615282 -1.669482 0.774708
H 2.616775 -1.667952 -0.774607

IIId
E = -471.522628
O 0.448038 -1.562697 -0.442158
Al -0.000910 -0.000170 1.106563
O 1.130212 1.169012 -0.441789
O -1.577130 0.393876 -0.443015
H 0.495291 -2.498682 -0.191895
H 1.175191 -1.403643 -1.064936
H -2.411184 0.820633 -0.191737
H -1.803262 -0.314958 -1.066145
H 0.629632 1.720017 -1.064444
H 1.917203 1.677321 -0.190475

IIIe
E = -471.577232
O 0.000179 1.519972 -0.340975
Al -0.000245 0.100289 0.611414
H -0.002843 2.447829 -0.096928
H 0.000045 -0.401285 2.084926
O 1.471193 -0.756519 -0.321994
H 2.061677 -1.458430 -0.010265
H 1.911688 -0.241759 -1.018121
O -1.471162 -0.756318 -0.321482
H -2.057238 -1.463435 -0.013355
H -1.911815 -0.243759 -1.019026

IVa
E = -547.974178
O -2.032607 -0.559851 -0.000796
Al -0.292871 -1.759222 0.000544
O 0.579837 0.065682 -0.000008
O -1.392047 2.101194 0.000249
H -2.072502 0.423643 -0.000504
H -2.929567 -0.922165 -0.001095
H 1.571380 0.194943 -0.000646
H 0.130595 0.934192 -0.001401
H -1.544843 2.670080 -0.768290
```

H -1.542270 2.667050 0.771534
O 3.180413 0.416113 -0.000356
H 3.755786 0.358542 -0.773916
H 3.753980 0.358500 0.774546

IVb
E = -547.972738
O -1.351752 0.525564 0.142568
Al -0.000144 -1.028677 0.000195
O 1.351706 0.525397 -0.142126
H -1.224747 1.355420 0.626620
H -2.339294 0.359568 0.075949
H 1.224621 1.354916 -0.626745
H 2.339219 0.359357 -0.075624
O 3.922753 0.099958 0.048294
H 4.490073 -0.343682 -0.595021
H 4.476271 0.312218 0.810565
O -3.922449 0.099566 -0.049000
H -4.490789 -0.341494 0.595170
H -4.475558 0.312619 -0.811330

IVc
E = -547.972488
O -0.943749 -1.330940 0.112279
Al -2.464601 0.000710 -0.414758
O -0.942760 1.330334 0.112749
O 1.250406 -0.000319 0.873764
H -0.036757 -1.077413 0.424587
H -1.030804 -2.293868 0.088498
H -1.028597 2.293379 0.089499
H -0.036372 1.075347 0.426015
H 1.531124 -0.000792 1.799125
H 2.085870 -0.000215 0.333449
O 3.456730 0.000132 -0.612859
H 3.995093 -0.771824 -0.829295
H 3.995232 0.772497 -0.827491

IVd
E = -547.967865
O -0.299746 -1.368610 -0.003848
Al 1.109408 -0.004769 -1.000101
O 2.111097 0.004560 0.953566
O -0.296071 1.367592 -0.011374
O -2.721948 0.002716 0.269136
H -0.243272 -2.314720 -0.200096
H -1.248006 -1.137879 0.094052
H 3.076051 -0.001721 1.037153
H 1.735395 0.005852 1.847415
H -1.245866 1.142831 0.084711
H -0.233516 2.312555 -0.211391
H -3.404028 -0.000642 -0.418208
H -3.205715 0.005659 1.107840

IVe
E = -548.029283
O 0.131811 1.396220 -0.298233
Al -0.827244 -0.010844 -0.648891
O 0.505015 -1.268079 -0.279654
O -1.873074 -0.116277 1.002776
H -0.068737 2.289301 -0.592148
H -1.818478 -0.448072 -1.769628
H -2.760208 -0.491618 1.104310
H -1.664215 0.433610 1.773493
H 0.555270 -2.193128 -0.548885
H 1.419296 -0.882051 0.036058
O 2.441436 0.047271 0.549876
H 2.067189 0.913233 0.303046
H 3.382559 0.046617 0.331208

TS1
E = -395.073697
O 0.000000 1.246804 0.000000
Al -1.340094 -0.313713 0.000000
O 1.560386 -0.827133 0.000000
H 0.921922 0.902200 0.000000
H -0.020803 2.216458 0.000000
H 2.018505 -1.198880 0.768555
H 2.018505 -1.198880 -0.768555

TS2
E = -395.003136
O -1.414133 0.547166 -0.000008
Al -0.133395 -0.666004 -0.000009
O 1.584434 0.321411 0.000006
H -1.812146 -0.888673 0.000097
H -1.950575 1.359225 0.000019
H 2.481868 -0.054677 0.000010
H 1.652580 1.293560 0.000004

TS3
E = -394.972771
o 1.221567 -0.641469 -0.085218
al 0.188700 0.728851 -0.114736
o -1.425181 -0.331685 0.012399
h 2.185829 -0.672806 -0.089657
h -2.357999 -0.071262 -0.046013
h -1.350680 -1.299204 0.097974
h 0.698657 0.353440 2.111819

I1, M=3
E = -394.957086
O -0.142696 0.646228 -0.000229
Al -1.730016 -0.327139 0.000013
O 2.195163 -0.221844 0.000042
H 0.825030 0.261475 0.000022
H -0.124680 1.616009 0.001368
H 2.684822 -0.510471 0.782393
H 2.685301 -0.509277 -0.782460

I2, M=3
E = -394.979485
O 1.548748 -0.450775 0.000108
Al -0.000100 0.759620 -0.000058
O -1.548788 -0.450448 0.000040
H 2.070099 -0.665621 0.790232
H 2.068918 -0.665574 -0.790808
H -2.068523 -0.667589 -0.790500
H -2.068874 -0.666495 0.790649

TS1, M=3
E = -394.946543
O 0.412441 1.098076 -0.098603
Al 1.167431 -0.657231 0.020646
O -1.744418 -0.264040 0.021915
H -0.578559 0.963974 -0.125711
H 0.648766 1.817188 0.509835
H -2.177547 -0.633647 -0.762426
H -2.413443 -0.275797 0.723401

TS2, M=3
E = -394.950151
O -1.594821 0.401347 -0.088885
Al -0.090677 -0.692567 0.008657
O 1.561022 0.369931 -0.027278

H -1.125150 0.352534 1.112047
H -1.593912 1.332259 -0.377785
H 1.845696 0.969898 0.683071
H 2.322565 0.178455 -0.600574

I4
E = -395.063762
O -1.646831 0.133008 -0.000000
Al 0.000000 0.063126 -0.000000
O 1.521127 -0.607477 0.000000
H -2.380317 0.753549 -0.000000
H 0.407387 2.015068 -0.000000
H 1.845935 -1.515093 0.000000
H 1.132632 1.721593 0.000000

I4, M=3
E = -394.949496
O -1.276994 0.365283 0.085175
Al 0.114088 -0.667377 -0.413239
O 0.968852 0.826724 0.128640
H -1.540038 0.637496 0.982646
H 0.670126 -1.913158 1.347930
H 0.992075 1.665921 -0.366292
H 0.859833 -1.250423 1.697294

I3
E = -395.099955
O -1.393755 -0.261865 -0.000000
Al 0.000000 0.676490 0.000000
O 1.215067 -0.779235 0.000000
H -2.330797 -0.046877 -0.000000
H 0.705459 2.056863 0.000000
H 2.185742 -0.786082 0.000000
H 0.869094 -1.689478 -0.000000

TS4
E = -395.039099
O -1.653279 -0.295623 -0.000020
Al -0.091821 0.250491 0.000008
O 1.549608 -0.325611 -0.000011
H -2.538888 0.079603 0.000100
H 0.821888 1.704899 -0.000015
H 1.557262 0.988864 -0.000008
H 2.182782 -1.059873 0.000073

I3, M=3
E = -394.971629
O 1.252306 -0.647188 -0.100526
Al 0.168420 0.741236 -0.079855
O -1.436648 -0.345453 0.020230
H 2.217040 -0.623891 -0.010740
H -2.356285 -0.087222 -0.150178
H -1.387665 -1.303402 0.186899
H 0.812185 0.319570 1.654509

TS4, M=3
E = -394.909875
o -0.722095 -1.160032 -0.088837
al 0.620766 -0.000003 -0.000021
o -0.722075 1.160043 0.088854
h -1.379040 -1.414515 0.582940
h 2.187276 -0.000009 -0.000033
h -1.379095 1.414488 -0.582865
h 4.054260 -0.000009 0.000098

H2O dis.
E = -318.625537
Al -0.000000 -0.000000 0.980754
O 0.000000 0.000000 -1.159061
H 0.000000 0.781657 -1.738654
H -0.000000 -0.781657 -1.738654

H2O dis, M=3
E = -318.497432
Al 0.000000 0.000000 0.880839
O -0.000000 -0.000000 -1.033081
H -0.000000 0.795895 -1.593127
H -0.000000 -0.795895 -1.593127

H2O dis 2
E = -318.603068
o 0.011964 1.023150 0.000000
al 0.011964 -0.608618 -0.000000
h -0.432973 1.874676 -0.000000
h 0.181733 -2.147839 0.000000

H2O dis 2, M=3
E = -318.483962
o -0.086361 0.975629 0.000000
al -0.086361 -0.703965 -0.000000
h -0.809780 1.618568 0.000000
h 2.623357 -0.272059 0.000000

H2 dis
E = -393.886340
O -1.640035 0.070656 -0.077840
Al 0.000024 -0.000425 0.050595
O 1.639939 -0.069366 -0.078803
H -2.403608 -0.384508 0.295126
H 2.404068 0.379717 0.300294

H2 dis, M=3
E = -393.780845
O 1.149071 -0.537334 -0.095510
Al -0.000139 0.837324 -0.000057
O -1.149128 -0.537204 0.095332
H 1.412675 -1.142661 0.622521
H -1.410406 -1.146245 -0.620360

H dis
E = -394.471870
O 1.239416 -0.612842 -0.000035
Al 0.210213 0.735630 -0.000034
O -1.400020 -0.327205 0.000534
H 2.201096 -0.670296 -0.000196
H -2.337154 -0.074405 -0.002396
H -1.311884 -1.298106 -0.000955

H2O
E = -76.423417
O 0.000000 0.117818 0.000000
H 0.764165 -0.471268 -0.000000
H -0.764165 -0.471274 -0.000000

H
E = -0.501657
H 0.000000 0.000000 0.000000

H2
E = -1.164101
H 0.000000 0.000000 0.380421
H 0.000000 0.000000 -0.380421

I2_S1

E = -394.776356
O 0.139992 0.653970 0.000064
Al 1.685043 -0.331404 -0.000242
O -2.150093 -0.206405 0.000233
H -0.826313 0.265693 0.000158
H 0.145238 1.613759 0.000206
H -2.571843 -0.575800 -0.775011
H -2.571834 -0.575920 0.775423

I5
E = -394.975349
O -1.382913 -0.022707 0.000018
Al 0.390424 -0.763086 -0.000017
O 1.178875 0.739946 0.000016
H -2.249961 -0.458160 0.000014
H -1.495760 0.955357 0.000048
H 2.119169 0.949218 0.000012
H -1.816659 2.735787 -0.000116

TS(S1) between I1 and I2
E = -394.765540
O -0.400574 1.076307 -0.100355
Al -1.161680 -0.641123 0.021597
O 1.731268 -0.258049 0.022759
H 0.581840 0.933123 -0.111860
H -0.655993 1.777337 0.504343
H 2.404647 -0.273201 0.705798
H 2.125799 -0.648723 -0.758268

TS5
E = -394.975590
al -0.173970 -0.839120 -0.000001
o -1.208682 0.532742 -0.000002
h -0.187937 2.280068 0.000003
o 1.356058 0.294960 0.000003
h -2.172925 0.593303 -0.000004
h 1.127479 1.264101 0.000004
h 2.315984 0.149470 0.000005

I5
E = -394.976940
O 1.380628 0.023886 -0.000017
Al -0.358242 -0.769697 -0.000004
O -1.195477 0.707030 0.000010
H 2.262710 -0.381565 -0.000027
H 1.458343 1.014164 -0.000013
H -2.141071 0.891006 0.000020
H 1.595960 2.635128 0.000127

TS6
E = -394.977681
O 1.402693 0.044545 0.000001
Al -0.302261 -0.680998 -0.000004
O -1.365224 0.625728 0.000003
H 2.226460 -0.476442 -0.000002
H 1.677432 1.102871 0.000007
H -2.326377 0.687210 0.000003
H 2.052130 2.177151 0.000013

I6
E = -395.052283
O 1.364525 -0.343291 0.033898
Al -0.244093 -0.024769 -0.037098
O -1.875925 0.162735 0.095331
H 2.218336 0.120832 -0.001578
H 3.867046 0.715354 -0.413203
H -2.648766 0.074754 -0.473689
H 3.827793 0.855511 0.336905

I5, M=3
E = -394.975349
O -1.382913 -0.022707 0.000018
Al 0.390424 -0.763086 -0.000017
O 1.178875 0.739946 0.000016
H -2.249961 -0.458160 0.000014
H -1.495760 0.955357 0.000048
H 2.119169 0.949218 0.000012
H -1.816659 2.735787 -0.000116

TS6, M=3
E = -394.940519
o -1.130660 -0.495269 -0.073712
al 0.234605 0.754270 0.045044
o 1.434276 -0.477920 -0.115515
h -1.284403 -1.246982 0.532213
h -2.681814 0.028367 -0.122088
h 1.933771 -1.037878 0.494990
h -3.446353 0.236498 0.023129

I6, M=3
E = -394.944463
O 1.001034 -0.316053 -0.377598
Al -0.649945 -0.708012 0.174813
O -0.910422 1.033512 -0.114348
H 1.785579 0.096618 0.048150
H 3.367540 0.940478 0.246553
H -0.708794 1.811371 0.435270
H 3.280062 0.616014 0.933023

TS5, M=3
E = -394.944403
o -1.315141 -0.466211 -0.057405
al -0.065129 0.791072 0.027655
o 1.450158 -0.420643 -0.021752
h -1.868642 -1.246863 0.961392
h -2.102582 -0.357824 -0.613827
h 1.382266 -1.330085 -0.361232
h 2.355501 -0.254322 0.287410
